# A Case of Fracture of 7th Cervical Vertebra

**Published:** 1871-06

**Authors:** J. W. Grosvenor

**Affiliations:** Lockport N. Y.


ART. III.—*A Case of Fracture of 7th Cervical Vertebra. Death on the 20th day.* By J. W. GROSVENOR, M. D., Lockport N. Y.

January 30th, 1871, I was called to L. W—, a rather large stoutly built man, about 45 years of age. Three quarters of an hour previously, he had fallen in a barn from a scaffold, a distance of fifteen feet. His head is supposed to have struck upon a wooden rail. I found a severe scalp wound, extending from near the outer corner of the right eyebrow to the posterior part of the right mastoid process. The line of the scalp wound was curved, the highest point of the curve reaching to near the parietal suture. He was reported to have lost much blood, pulseless and extremities cold. He complained of severe pain on the back of his neck, which was aggravated by the slightest movement of his head in any direction. He could move his head slightly of his own accord; could raise his arms to his head; had no control whatever over his lower extremities. Examining with the point of a pin no sensation was discovered in the lower extremities, none in the right hand, none in the trunk, inferior to a horizontal line drawn about three inches below the top of sternum. A slight sense of feeling in the left hand. The power of sensation remained in both arms, head, and all that part of the trunk, superior to the horizontal line above mentioned. On 5th day, sensation remained in both arms, but patient had only slight control over them; could carry his hands to his head with an irregular jerking movement. From this day until death he suffered occasionally from dyspnœa.

Electricity being applied on the 6th day, battery was not felt except when applied to arms and head.

On the 8th day patient had lost complete control of right arm and hand, but still retained partial use of left arm and hand. He could move his head from side to side with less pain.

On the 11th day, complete anæsthesia existed in both hands and wrists; also on outside of both arms as high as elbows. On front side of both arms sensation commenced two or three inches above the wrists. In other parts of the body power of sensation remained as before described. From this date until death no change was observed in the power of sensation in any part of the system. Retention of urine existed at the outset and continued throughout the case, necessitating the use of the catheter twice daily. Bowels were easily moved by castor oil, though unaffected by enemas of simple water.

After re-action took place on the day following the accident, the pulse assumed fulness, regularity, and sixty beats per minute, and continued thus until the 10th day, when it rose to sixty-six beats per minute. From the 10th day the pulse gradually became weaker and increased in frequency until death, but at no time rose above seventy-two beats per minute.

The tongue was, most of the time, dry and covered with a brown fur.

On the 16th day was observed on the nates, an inflammatory condition, which passed into gangrene, doubtless the result in part of a bruise at the lower extremity of the spinal column, which was received at the time of the injury, and in part of a constant supine position.

During the last two days of life the patient had an occasional aberration of mind. Throughout his sickness there existed a difficulty in the articulation of words—a thickness of speech.

Treatment consisted, at first, of alcoholic stimulants, until re-action came on, anodynes to procure rest and sleep, quinine as a tonic, animal broths for nourishment.

Autopsy, 30 hours after death. Body rigid and extremely bloated so as to assume twice its natural size, cellular tissue filled with gas so that percussion gave a resonant sound. Examined only upper part of spinal column. On removing 4th, 5th, 6th and 7th cervical vertebra, a fracture was found in the superior part of the body of the 7th. Two pieces were split off horizontally from the right side of the body of the vertebra, one was about half an inch thick at its outer edge and very thin at its inner edge, and covered about half of the upper surface of the body of the vertebra. The other fragment was not larger than a wafer of ordinary size, and was chipped off from the upper surface of the left side of the body of the vertebra. These fractures permitted the 7th cervical vertebra to fall backward about a third of an inch, and press upon the spinal cord. Membranes of spinal cord intact, cord itself for half an inch below and half an inch above the fractures was liquified to the consistency of thick cream.

This case was chiefly remarkable for the length of time the patient survived after the accident.

